# LC3B, mTOR, AMPK Are Molecular Targets for Neoadjuvant Chemotherapy in Gastric Cancers

**DOI:** 10.3390/cimb44070190

**Published:** 2022-06-26

**Authors:** Liudmila V. Spirina, Alexandra V. Avgustinovich, Olga V. Bakina, Sergey G. Afanas’ev, Maxim Yu. Volkov, Amina Y. Kebekbayeva

**Affiliations:** 1 Biochemistry and Molecular Biology Department, Siberian State Medical University, 2, Moskovsky Trakt, Tomsk 634050, Russia; ovbakina@ispms.tsc.ru (O.V.B.); kebekbayevaa58@gmail.com (A.Y.K.); 2 Tomsk National Research Medical Center of the Russian Academy of Sciences, Cancer Research Institute, 5 Kooperativny Street, Tomsk 634050, Russia; aov862@yandex.ru (A.V.A.); afanasievsg@oncology.tomsk.ru (S.G.A.); dok75-75@mail.ru (M.Y.V.); 3 Institute of Strength Physics and Materials Science of the Siberian Branch of the Russian Academy of Sciences, 2/4, pr. Akademicheskii, Tomsk 634055, Russia

**Keywords:** gastric cancer, LC3B, mTOR, AMPK, NACT

## Abstract

Autophagy plays a dual role in oncogenesis processes. On one hand, autophagy enhances the cell resistance to oncogenic factors, and on the other hand, it participates in the tumor progression. The aim of the study was to find the associations between the effectiveness of the FLOT regimen in resectable gastric cancers (GCs) with the key autophagy-related proteins. **Materials and Methods:** The study included 34 patients with morphologically verified gastric cancer. All patients had FLOT neoadjunvant chemotherapy (NACT) (fluorouracil, leucovorin, oxaliplatin, and docetaxel) followed by gastrectomy. The studied tissue material was the non-transformed and tumor tissues obtained during diagnostic video gastroscopy in patients before the start of the combined treatment and after surgical treatment, frozen after collection. The LC3B, mTOR, and AMPK expression was determined by real-time PCR. The content of the LC3B protein was determined by Western blotting analysis. **Results:** The mRNA level and the content of the LC3B protein were associated with the tumor stage and the presence of signet ring cells. The AMPK mRNA level was increased in patients with the T4N0-2M0 stage by 37.7 and 7.33 times, which was consequently compared with patients with the T2N0M0 and T3N0-1M0 stages. The opposite changes in the mTOR and AMPK in the GCs before anti-cancer therapy were noted. The tumor size and regional lymph node affections were associated with a decrease in the mTOR mRNA level. A decrease in the mTOR expression was accompanied by an increase in the AMPK expression in the GCs. The mTOR expression was reduced in patients with a cancer spreading; in contrast, AMPK grew with the tumor size. There was an increase in the LC3B expression, which can probably determine the response to therapy. An increase in LC3B mRNA before the start of treatment and the protein content in cancers after NACT with a decrease in therapy effectiveness was recorded. There was an increase in the protein level in patients with partial regression and stabilization by 3.65 and 5.78 times, respectively, when compared with patients with complete tumor regression was noted. **Conclusions:** The anticancer effectiveness in GCS is down to the LC3B, mTOR, and AMPK expression. These were found to be entire molecular targets affecting the cancer progression and metastasis as well as the NACT effectiveness.

## 1. Introduction

Gastric cancer (GC) is one of the most prevalent malignant types in the world, with a poorly understood carcinogenesis at the molecular and genetic level [1]. GC is one of the most common cancers and presents an urgent global problem and belongs to the third most lethal tumors worldwide (8.3% of all cancer deaths are attributable to gastric cancer) [2]. Multiple mechanisms are known to participate in the GC initiation and progression [3]. The key and poorly understood pathway in oncogenesis is associated with autophagy [4]. The activation of “self-eating” is believed to be initiated by Helicobacter pylori infection, which promotes tumorigenesis of the gastric mucosa [5] and protects cells from apoptosis [6,7].

Autophagy is associated with the processes of cell survival, its death. It is a vital intracellular homeostatic process through which defective proteins and organelles are degraded and recycled under starvation, hypoxia, or other specific cellular stress conditions [8]. This is known to affect the metastatic processes of gastric cancer by acting on a wide range of molecular targets including the degradation of the extracellular matrix, the development of the epithelial–mesenchymal transition, tumor angiogenesis, and modification of the tumor microenvironment [9].

Autophagy plays a dual role in oncogenesis [4]. On one hand, it increases the cell resistance to oncogenic factors; on the other hand, it participates in the processes of tumor progression and the formation of resistance to antitumor treatment [6,10]. It has been shown that autophagy initiation is correlated with the aggressive course of the disease and its poor prognosis [11].

The proteins associated with autophagy include a protein complex that plays a decisive role at all stages of autophagosome development, for example, the autophagy-associated protein Atg13 (ULK1), Beclin—1, vacuolar protein sorting 34 (VPS34), VPS15, Atg14, and associated with microtubule protein 1A/1B-light chain 3—(LC3B). High levels of LC3B are found in gastric cancer cells [12] and can predict the disease outcome [13].

The molecular cascades that regulate autophagy are many and varied. Research efforts are focused on the serine/threonine protein kinases, AMP-activated protein kinase, (AMPK) and the rapamycin-inhibited kinase of mammals (mTOR) [14,15]. There is also evidence that AMPK is associated with the resistance to anti-cancer treatment in GCs [16]. The role of the mTOR kinase is also diverse. It integrates various signaling pathways including the AKT/mTOR, activated under the influence of growth factors and mitogens [17,18,19].

The list of molecular biomarkers that can be used for prognostic purposes varies annually [20]. Currently, there are data on markers predicting the effect of neoadjuvant therapy in GC. It is known that the expression level of the AKT gene can predict the impact of chemotherapy in GC [21,22]. It is believed that a complex of molecular indicators is involved in both anti-cancer process and tumorigenesis [23]. The AKT/mTOR signaling cascade triggers sensitivity to treatment [24] and autophagy [17]. The effect of therapy based on 5-fluorouracil and cisplatin is known to be associated with changes in the expression profiles of the intracellular signaling cascade components (the AKT/mTOR signaling cascade and autophagy markers) [25] and can predict the effect of the anti-cancer treatment [26,27]. In general, the role of biological indicators associated with autophagy in GC progression and response to therapy is still unclear.

To date, surgical therapy is the main approach in GC treatment. Its subclinical hematogenous and lymphogenous metastases have become the main reasons for the unsatisfactory long-term results for GCs. In locally advanced, resectable gastric or gastro-esophageal junction adenocarcinoma, perioperative FLOT improved overall survival compared to the ECF/ECX (either three pre-operative and three postoperative 3-week cycles of 50 mg/m^2^ epirubicin and 60 mg/m^2^ cisplatin on day 1 plus either 200 mg/m^2^ fluorouracil as continuous intravenous infusion or 1250 mg/m^2^ capecitabine orally on days 1 to 21) [27,28].

The aim of the study was to find the associations between the effectiveness of the FLOT regimen in resectable gastric cancers (GCs) with the key autophagy-related proteins.

## 2. Materials and Methods

The patients were admitted to the Cancer Research Institute, Tomsk National Research Center, Russian Academy of Medical Sciences, Tomsk, Russian Federation. Tumor response to therapy was evaluated according to the RECIST 1.1 criteria. The study included 34 patients with GC who received three courses of FLOT neoadjunvant chemotherapy (NACT) (fluorouracil, leucovorin, oxaliplatin, and docetaxel). The exclusion criteria were: previous special treatment, cardiac localization of the tumor, the distant metastases, primary multiple synchronous and metachronous process (except for basal cell skin cancer), clinically significant comorbidities, individual intolerance to chemotherapy components, and complicated forms of gastric cancer (cachexia, decompensated pyloric stenosis, ongoing gastric bleeding requiring emergency surgery, tumor perforation).

The clinical and morphological characteristics of gastric cancer patients are presented in Table 1. The age of patients ranged from 36 to 69 years; the average age was 57.1 ± years. There were 26 men (76%), and eight women (24%). In most cases, 31 (91%), adenocarcinoma of varying degrees of differentiation occurred; undifferentiated or cricoid-cell GC was diagnosed in three (9%) patients. According to the localization of the tumor, the patients were divided as follows: the body of the stomach—12 (48%), antrum—7 (28%), subtotal lesion—6 (24%).

The TNM classification (UICC) 7th revision was applied for staging. cT2N0 had seven (20%) people, cT3N0 had nine (25%), cT4N0 had one (4%), cT3N1 had eight (23%), cT4N1 had four (12%), cT4N2 had one (4%), and cT4N3 had three (12%).

Before treatment, all patients underwent a comprehensive examination including X-ray/computed tomography of the chest, video-gastroduodenoscopy with biopsy, endoscopic ultrasonography, ultrasound of the pelvic organs (in women), and computed tomography of the abdominal organs with contrast enhancement. Laparoscopy was used to exclude the peritoneal dissemination of gastric cancer.

At the preoperative stage, all patients underwent eight courses of chemotherapy according to the FLOT scheme (docetaxel 50 mg/m^2^, oxaliplatin 85 mg/m^2^, leucovorin 200 mg/m^2^, and 5-FU 2600 mg/m^2^ for 48 h) with an interval between courses of 14 days.

The assessment of the effectiveness of preoperative chemotherapy (after 3, 6, and 8 courses) was carried out according to the RECIST 1.1 criteria (complete or partial regression, stabilization, progression).

The study of chemotherapy tolerance was carried out using the NCIC common toxicity criteria grading system. Radical surgery was performed 4–8 weeks after the completion of chemotherapy. The volume of surgical intervention performed depended on the tumor localization. The frequency and nature of postoperative complications were presented on the Clavien–Dindo scale.

Pathologic assessment of tumor regression after preoperative chemoradiotherapy was performed using the Mandard scale. Biopsy post-operative samples of normal gastric and tumor tissues were used for investigation. Samples were reviewed separately by two independent pathologists. Tissues were frozen and stored at t = 80 °C.

The Local Committee of Medical Ethics in Cancer Research Institute, Tomsk National Research Medical Center of the Russian Academy of Sciences approved this work, Minute No. 5, dated 24 April 2019.

### 2.1. RNA Extraction

The tumor samples were incubated in RNAlater solution (Ambion, Austin, TX, USA) for 24 h at + 4 °C and then stored at −80 °C. Total RNA was extracted using RNeasy Mini Kit (Qiagen).

RT-qPCR was performed according to [28]. PCR was conducted in 25 μL reaction volumes containing 12.5 μL BioMaster HS-qPCR SYBR Blue (2X) (“Biolabmix” Russia) and 300 nanoM of each of the following primers: LC3B: F 5’-CCCAAACCGCAGACACAT-3’, R 5’-ATCCCACCAGCCAGCAC-3’; m-TOR: F 5’-CCAAAGGCAACAAGCGAT-3’, R 5’-TTCACCAAACCGTCTCCAA-3’; AMPK: F 5’-AAGATGTCCATTGGATGCACT-3’, R 5’-TGAGGTGTTGAGGAACCAGAT-3’; GAPDH: F 5’-GGAAGTCAGGTGGAGCGA-3’, R 5’-GCAACAATATCCACTTTACCAGA-3’.

A pre-incubation at 95 °C for 10 min was to activate the Hot Start DNA polymerase and denature the DNA, and was followed by 45 amplification cycles of 95 °C denaturation at 95 °C for 10 s, and 60 °C annealing at 60 °C for 20 s (iCycler iQ™, BioRad, Hercules, CA, USA).

The fold changes were calculated by the ΔΔCt method (the total ΔΔCt = fold of cancerous/normal tissue gene level) using normal tissue. A ratio of specific mRNA/GADPH (GADPH as a respective control) amplification was then calculated.

### 2.2. Determination of LC3B Content

Electrophoresis SDS-PAGE (Laemmli) was used. The protein was transferred to 0.2-/xm pore-sized PVDF membrane (GE Healthcare, Chalfont Saint Giles, UK), either at 150 mA or 100 V for 1 h by using a Bio-Rad Mini Trans-Blot electrophoresis cell. The membrane was incubated in a 1:2500 dilution of monoclonal mouse anti-human LC3B (Affinity Biosciences, Cincinnati, OH, USA) at 4 °C overnight.

PVDF samples were incubated in an Amersham ECL Western blotting detection analysis system (Amersham, Kingsport, TN, USA). The results were standardized using the beta-actin expression in a sample and were expressed in percentages to the protein content in non-transformed tissues. The level of protein in normal gastric tissue was indicated as 100%.

### 2.3. Statistical Analysis

Statistical analysis was performed using SPSS 19.0 software. Data were expressed as median and ranges. The Mann–Whitney test was used to compare the differences in the mean values in two independent groups. Nonparametric one-way ANOVA on ranks was carried out to test whether the samples originated from the same distribution, which was used to compare two or more independent samples of equal or different sample sizes. The median test and Kruskal–Wallis test were applied. Nonparametric correlation analysis was performed, and the Spearmen coefficient was calculated.

## 3. Results

### 3.1. FLOT Regimen in GC Patients, Its Effectiveness

All patients completed the planned courses of preoperative chemotherapy (100%). The chemotherapy side effects did not require treatment interruption. Table 2 represents the list of the revealed side effects. Nausea was found in 23 patients (92%), peripheral neuropathy I–II degree in 15 (60%), neutropenia I–II degree in 12 (48%), vomiting in 10 (40%), diarrhea in five (20%), and bronchospasm in one (4%).

Partial tumor regression was detected in 20 (80%) patients. Stabilization was registered in five cases (20%). There were no cases of complete response and cancer progression. Surgical treatment was carried out (R0) in all patients. The subtotal gastric resection was performed in 11 patients (55%) and gastrectomy in nine patients (45%). There were no cases of postoperative mortality. Most of the patients had therapeutic pathomorphosis TRG2—11 (44%), TRG3—10 (40%), TRG4—3 (12%), and TRG5—1 (4%) (Table 3).

After preoperative chemotherapy, a decrease in the clinical stage according to the T and N indices was noted in 13 (52%) patients and in 11 (44%) patients, consequently. An increase in the clinical stage according to the N criterion was noted in one patient.

The efficacy and safety of preoperative FLOT regimen chemotherapy in patients with resectable GCs was verified. The stabilization in GC patients was found in 92% of cases. A partial response was found in 50% of patients. We did not detect any cases of GCs progression.

### 3.2. Molecular Targets in GCs

#### 3.2.1. The Expression of LC3B, mTOR, AMPK, and the Content of LC3B Protein in Gastric Cancer Tissue Depends on the Disease Clinical and Morphological Parameters

We found an elevated LC3B mRNA level before the start of neoadjuvant chemotherapy (NACT) accompanied by the increase in tumor size (Table 4). The mTOR and AMPK expression in GC tissue were associated with the clinical and morphological indicators before treatment. The AMPK mRNA level increased in patients with the T_4_N_0–2_M_0_ stage by 37.7 and 7.33 times when consequently compared with patients with the T_2_N_0_M_0_ and T_3_N_0–1_M_0_ stages, respectively. The affect of the regional lymph nodes was associated with a decrease in the mTOR mRNA level. The LC3B protein content, assessed after NACT, was related to the tumor size, the lesions of regional lymph nodes, and the presence of signet ring cells.

The relationship between the LC3B expression and its protein content after NACT with the GC grade and histological type was revealed. In cancers with the signet ring cell, a decrease in the LC3B expression was detected by 8.47 times. The autophagy protein was also decreased by 4.7 times after NACT in patients with low-grade adenocarcinoma (Table 5). However, the mTOR and AMPK mRNA levels were not related to the GC grade and the presence of the signet ring cell.

**Figure 1:**
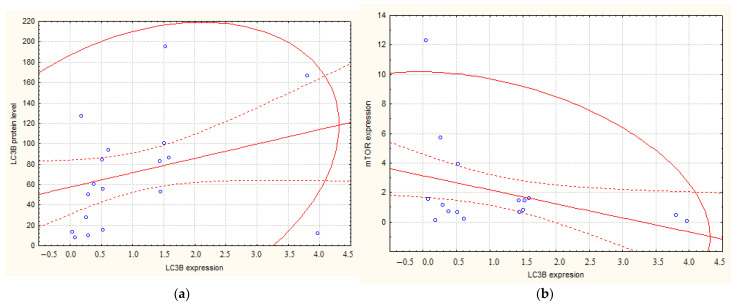
The scatter plots between the LC3B expression and corresponding protein level (**a**) and mTOR expression (**b**). Note: The corresponding protein levels accompanied increased LC3B expression. In this case, mTOR was inhibited, which is probably associated with resistance to NACT.

There was a correlation between the level of LC3B mRNA before treatment and the protein content after NACT, as measured by Western blotting analysis (r = 0.43; *p* = 0.013) (Figure 1a). A negative relationship was also revealed between the studied parameter and mTOR expression (r = − 0.38; *p* = 0.03) (Figure 1b).

#### 3.2.2. Changes in the LC3B, mTOR, AMPK Expression in Tumor Tissue after the NACT

**Figure 2:**
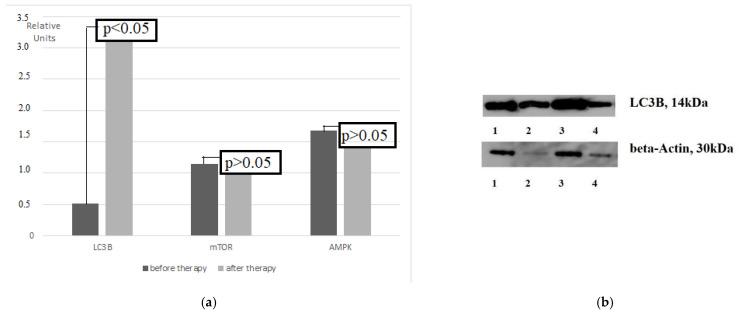
The expression of LC3B, mTOR, and AMPK in the gastric tumor tissue before and after NACT (**a**) and Western blotting of LC3B (**b**). Note: A—An increase in the LC3B mRNA level was revealed during therapy with the FLOT regimen in patients with GCs; B—Western blot; 1, 3—LC3B protein in cancers; 2, 4—LC3B protein in non-transformed tissues.

An increase in the LC3B expression by 5.94 times was obtained during the combined anti-cancer therapy (Figure 2). Simultaneously, the expression of autophagy regulators, kinases mTOR, and AMPK after treatment did not change.

#### 3.2.3. Relationship of LC3B, mTOR, AMPK Expression, and LC3B Protein Content in GC Tissue with the Effectiveness of NACT

**Figure 3:**
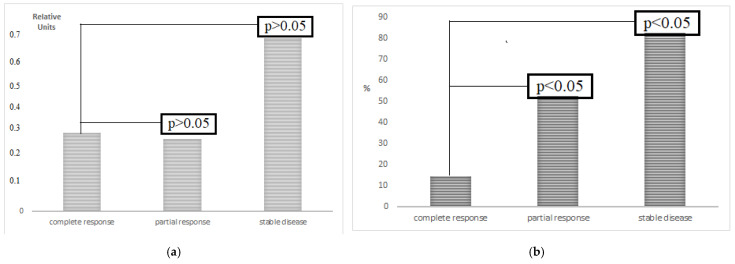
The expression (**a**) and content of the LC3B protein (**b**) in the gastric cancer tissue versus therapy efficacy. **Note:** A—LC3B expression in GCs depends on the response to the therapy. The level of the indicator is not related to the effect of therapy: Kruskal–Wallis test: *p* < 0.05; Median test: *p* > 0.05; B—protein level in % relative to unchanged tissue (100%) in the tissue of patients after NACT, depending on the effectiveness of the therapy. The LC3B protein content is associated with the treatment effect: Kruskal–Wallis test: *p* < 0.05; Median test: *p* < 0.05.

The LC3B mRNA level in GCs before therapy was not associated with the response to treatment. However, a relationship between the LC3B content after NACT and the therapy’s effectiveness in cancers was found. An increase in the protein level by 3.65 and 5.78 times as a consequence was noted in patients with partial response and stabilization, respectively, when compared to patients with complete response (Figure 3). The data obtained indicate the role of autophagy in the GCs resistance to therapy.

The search for predictive molecular markers that determine the behavior of cancers is promising. The mTOR and AMPK expression are considered as selective markers in cancer response prediction. Table 6 presents the data on the relationship between the mRNA level of the studied markers and the effectiveness of the anti-cancer therapy. A decrease in the mTOR level before treatment in a tumor was found in patients with a partial response, complete response, and cancer progression. In this case, the expression of AMPK had an undulating character of changes. The pronounced differences, an 8- and 5-fold increase in the indicator, were observed in patients with disease progression compared with patients with partial regression.

## 4. Discussion

The variable anti-tumor response to NACT in patients with GCs was demonstrated once again. Despite the modern chemotherapeutical regimen and application approaches, it was shown that the heterogeneity in tumor response to anti-cancer therapy is associated with the biological characteristics of the tumor as well as autophagy activation.

The difference in the clinical and pathological stages in GC patients was noted. GCs with I–IB and IIB pathological stages after the NACT had signs of autophagy induction. Growth in the LC3B and AMPK expression was correlated with the increase in pTNM. It is known that the high content of LC3B protein in GCs is one of the cancer progression markers [12] that affect the patient’s outcome [13]. This study showed that LC3B expression was associated with tumor size and cancer spreading. The found data showed the involvement of autophagy in the oncogenesis that was followed by the patients prognosis. The highest level of “self-digestion” is reached in most spreading tumors. As the biology approved mechanism of cancer protection, autophagy and its marker LC3B determine the inner origin of the ineffective anti-cancer treatment.

The significant clinical features and morphological signs of aggressive behavior including low-grade cancers, signet ring cells were associated with an increase in the LC3B mRNA level. The main autophagy regulator, mTOR, was found to be reduced in patients with a cancer spreading and in HER2 positive tumors [20]. In contrast, AMPK increased in cancers with a higher tumor size. Unfavorable cancers were associated with a low LC3B expression before the onset of NACT, leading to the drop in the protein content after NACT. Aggressive cancer behavior was revealed to be dependent on the onset of autophagy and its regulators.

An increase in the LC3B expression was found as a result of effective anti-cancer therapy. This fact confirms the role of high LC3B content in unfavorable patient outcomes and poor response to the therapy [12,13]. An increase in the autophagy’s protein content with a decreased response to the treatment were recorded. The FLOT therapy based on 5-fluorouracil and cisplatin is associated with a change in the intracellular signaling cascades expressional profiles [27], thus predicting the anti-cancer effect [28]. Currently, there is lack of data about markers that could predict the NACT response in GCs [10,18,23,24], highlighting the molecular features of cancer sensitivity with the involvement of the autophagy-related route [16].

Critical regulators of autophagy include AMPK and mTOR [14,18,19]. The AMPK activation may influence the effectiveness of therapy [16] including the resistance to chemotherapy in GCs [16]. The opposite changes in the AMPK expression in the GCs before the anti-tumor therapy were noted. An increase in the AMPK expression was accompanied by a decrease in the mTOR expression.

The predictive molecular factors search for the GC response to NACT significantly affects the patient’s outcome. The complete response had no definite marker that influenced the effectiveness of the NACT. Changes in the mTOR expression and LC3B were found in patients with partial response, verifying the involvement of the biological factors in the behavior of aggressive cancers. The cancer progression affected NACT with the most pronounced changes and modification compared with the partial response. Even though multiple mechanisms are known to be responsible for the anti-cancer treatment benefits, autophagy regulation belongs to the most powerful processes that could explain the biology of variable response to the therapy in GC patients.

The present study presents a point of view showing the role of the biological characteristics of cancer in predicting the response to therapy. The powerful protective effect of autophagy in preventing tumor development during tumor progression is being altered. Already activated molecular mechanisms in the tumor trigger the resistance to treatment and are provoked by the NACT. The biological behavior modification with the growth in tumor aggressiveness is a consequence of the use of anti-cancer therapeutical agents. Chemotherapy interventions need to consider both the early signs of a poor cancer prognosis and the molecular-based effects of treatment.

## 5. Conclusions

A partial response was prevalent in most of the cases in the GC patients treated with the FLOT NACT regimen. The molecular features in tumors underlie the anti-cancer effectiveness. Therefore, we found a link between autophagy activation and therapy response.

The impact of the FLOT regimen on autophagy is a key regulatory target for effective therapy. The complex change in molecular markers was shown in GCs associated with aggressive biological and clinical features. This trend needs further investigation. The biological behavior modification is a significant prognostic factor. We found biomarkers predicting the partial response in GC patients down to the treatment effectiveness.

## Figures and Tables

**Table 1:** The clinical and morphological characteristics of the GC, *n* (%).

Indicator	*n* (%)
ECOG	
0	31 (92%)
1	3 (8%)
Gender	
male	26 (76%)
female	8 (24%)
Histology	
High differentiated	1 (3%)
Moderate differentiated	10 (28%)
Low-differentiated	20 (60%)
Non-differentiated	1 (3%)
Signet ring cell	2 (5%)
Cancer location	
body	16 (48%)
antral d	10 (28%)
Subtotal lesions	8 (24%)
cTN	
T2N0	7 (20%)
T3N0	9 (25%)
T4N0	1 (4%)
T3N1	8 (23%)
T4N1	4 (12%)
T4N2	1 (4%)
T4N3	4 (12%)

**Table 2:** The toxicity of preoperative chemotherapy, *n* (%).

Side Effects	*n* (%)
Nausea	23 (92%)
Peripheral neuropathy of I–II degree	15 (60%)
Grade I–II neutropenia	12 (48%)
Vomiting	10 (40%)
Diarrhea	5 (20%)
Bronchospasm	1 (4%)

**Table 3:** The degree of therapeutic pathomorphosis, *n* (%).

Pathomorphosis	*n* (%)
TRG1	---
TRG2	11 (44%)
TRG3	10 (40%)
TRG4	3 (12%)
TRG5	1 (4%)

**Table 4:** The LC3B, mTOR, AMPK expression, and LC3B protein content in the GCs after the NACT depending on the tumor size and regional lymph node involvement.

	T_2_N_0_M_0_ (*n* = 7)	T_3_N_0–1_M_0_ (*n* = 9)	T_4_N_0–2_M_0_ (*n* = 18)	T_2_N_0_M_0_ (*n* = 17)	T_2-3_N_1_M_0_ (*n* = 11)	T_3–4_N_2_M_0_ (*n* = 6)
LC3B expression, Relative Units	0.36 (0.19; 0.53)	0.38 (0.30; 0.62)	1.45 (0.08; 1.59)	0.30 (0.26; 0.53)	1.44 (0.38; 1.59)	1.50 (0.80; 1.65)
	Kruskal–Wallis test: *p* < 0.05; Median Test: *p* < 0.05			Kruskal–Wallis test: *p* < 0.05; Median Test: *p* < 0.05		
mTOR expression, Relative Units	0.97 (0.97; 1.35)	0.36 (0.19; 1.15)	0.99 (0.44; 1.60)	1.28 (0.66; 3.93)	0.66 (0.33; 1.22)	0.24 (0.09; 0.95) #
	Kruskal–Wallis test: *p* > 0.05; Median Test: *p* > 0.05			Kruskal–Wallis test: *p* < 0.05; Median Test: *p* < 0.05		
AMPK expression, Relative Units	0.07 (0.00; 1.16)	0.36 (0.02; 0.63)	2.64 (1.36; 7.95) *, **	0.63 (0.11; 1.45)	1.21 (0.27; 3.66)	1.39 (0.65; 14.94)
	Kruskal–Wallis test: *p* < 0.05; Median Test: *p* < 0.05			Kruskal–Wallis test: *p* > 0.05; Median Test: *p* > 0.05		
LC3B protein level, % to the normal tissues	126.75 (55.70; 240.77)	50.00 (11.98; 60.55) *	83.00 (13.00; 100.35)	27.3 (13.00; 55.00)	86.54 (60.55; 126.75) #	7.72 (2.54; 7.80) #, ##
	Kruskal–Wallis test: *p* < 0.05; Median Test: *p* < 0.05			Kruskal–Wallis test: *p* <0.05; Median Test: *p* < 0.05		

Note: *—the significance of differences in comparison with patients with tumor size T_2_N_0_M_0_, *p* < 0.05; **—the significance of differences in comparison with patients with tumor size T_3_N_0_M_0_, *p* < 0.05; #—the significance of the differences compared with patients with the stage of the disease T_2_N_0_M_0_, *p* < 0.05; ##—the significance of differences compared with patients with the stage of the disease T_2–3_N_1_M_0_, *p* < 0.05.

**Table 5:** The LC3B, mTOR, AMPK expression and the LC3B protein content in GCs depending on the grade and the signet ring cell detection.

	High-Differentiated Adenocarcinoma (*n* = 5)	Moderately-Differentiated Adenocarcinoma (*n* = 17)	Low-Differentiated Adenocarcinoma (*n* = 5)	Signet Ring Cell Carcinoma (*n* = 7)
LC3B expression, Relative Units.	1.45 (1.20; 1.65)	0.53 (0.32; 0.76)	1.44 (1.19; 3.80)	0.17 (0.08; 0.26) *
	Kruskal–Wallis test: *p* < 0.05; Median Test: *p* < 0.05			
mTOR expression, Relative Units.	0.64 (0.35; 0.85)	3.93 (2.89; 3.99)	0.97 (0.19; 1.60)	0.58 (0.20; 3.61)
	Kruskal–Wallis test: *p* > 0.05; Median Test: *p* > 0.05			
AMPK expression, Relative Units	0.11 (0.89; 0.15)	1.16 (1.00; 1.20)	1.01 (0.02; 4.06)	0.88 (0.45; 1.38)
	Kruskal–Wallis test: *p* > 0.05; Median Test: *p* > 0.05			
LC3B protein level, % to the normal tissues	52.50 (32.50; 86.70)	35.70 (15.70; 55.70)	83.00 (13.50; 126.70)	17.50 (7.72; 27.30)*
	Kruskal–Wallis test: *p* > 0.05; Median Test: *p* > 0.05			

Note: *—the significance of differences in comparison with patients with low-differentiated cancers, *p* < 0.05.

**Table 6:** The expression of mTOR and AMPK in the gastric tumor tissue depending on the effectiveness of anti-cancer therapy.

Indicator, Relative Units.	Complete Response (*n* = 4)	Partial Response (*n* = 20)	Stable Disease(*n* = 7)	Progressive Disease (*n* = 3)
mTOR	8.11 (3.93; 12.30)	0.64 (0.13; 0.99) *	0.69 (0.19; 1.46) *	0.75 (0.22; 1.28) *
	Kruskal-Wallis test: *p* < 0.05; Median Test: *p* < 0.05			
AMPK	6.30 (1.16; 11.45)	0.63 (0.11; 1.45)	1.13 (0.01; 2.16)	5.36 (2.77; 7.95) **
	Kruskal-Wallis test: *p* < 0.05; Median Test: *p* < 0.05			

Note: *—significance of differences in comparison with patients with complete response, *p* < 0.05; **—the significance of differences in comparison with patients with partial response, *p* < 0.05.
